# Malignant A-to-I RNA editing by ADAR1 drives T-cell acute lymphoblastic leukemia relapse via attenuating dsRNA sensing

**DOI:** 10.21203/rs.3.rs-2444524/v2

**Published:** 2023-06-16

**Authors:** Maria Rivera, Haoran Zhang, Jessica Pham, Jane Isquith, Qingchen Jenny Zhou, Roman Sasik, Adam Mark, Wenxue Ma, Frida Holm, Kathleen M Fisch, Dennis John Kuo, Catriona Jamieson, Qingfei Jiang

**Affiliations:** 1 Division of Regenerative Medicine, Department of Medicine, University of California, San Diego, La Jolla, CA, USA; 2 Moores Cancer Center, La Jolla, CA 92037, USA; 3 Center for Computational Biology & Bioinformatics (CCBB), University of California, San Diego, La Jolla, 92093-0681.; 4 Department of Women’s and Children’s Health, Division of Pediatric Oncology and Surgery, Karolinska Institutet, Sweden; 5 Department of Obstetrics, Gynecology & Reproductive Sciences, University of California, San Diego, La Jolla, CA; 6 Division of Pediatric Hematology-Oncology, Rady Children’s Hospital San Diego, University of California, San Diego, CA

**Keywords:** ADAR1, RNA editing, T-ALL

## Abstract

Leukemia initiating cells (LICs) are regarded as the origin of leukemia relapse and therapeutic resistance. Identifying direct stemness determinants that fuel LIC self-renewal is critical for developing targeted approaches to eliminate LICs and prevent relapse. Here, we show that the RNA editing enzyme ADAR1 is a crucial stemness factor that promotes LIC self-renewal by attenuating aberrant double-stranded RNA (dsRNA) sensing. Elevated adenosine-to-inosine (A-to-I) editing is a common attribute of relapsed T-ALL regardless of molecular subtypes. Consequently, knockdown of ADAR1 severely inhibits LIC self-renewal capacity and prolongs survival in T-ALL PDX models. Mechanistically, ADAR1 directs hyper-editing of immunogenic dsRNA and retains unedited nuclear dsRNA to avoid detection by the innate immune sensor MDA5. Moreover, we uncovered that the cell intrinsic level of MDA5 dictates the dependency on ADAR1-MDA5 axis in T-ALL. Collectively, our results show that ADAR1 functions as a self-renewal factor that limits the sensing of endogenous dsRNA. Thus, targeting ADAR1 presents a safe and effective therapeutic strategy for eliminating T-ALL LICs.

## INTRODUCTION

T-cell acute lymphoblastic leukemia (T-ALL) is an aggressive hematological malignancy that frequently occurs in children, adolescents, and young adults. Approximately 10–20% of T-ALL patients will experience relapse months or years following remission and will often become refractory to further treatments ^1,2^. The survival of relapsed/refractory patients is very poor, with an overall survival rate of less than a 25% overall survival rate ^3^. Relapsed patients often have enriched pools of leukemia initiating cells (LICs) with enhanced pro-survival and self-renewal capacity, suggesting a potential vulnerable population for effective targeted therapies with less toxicity ^4–6^.

An emerging research topic in LIC biology is the identification of RNA modifying enzymes that may cooperate with genetic lesions to provide advantages in important LIC functions ^7^. ADAR enzymes catalyze the transition of adenosine (A) to inosine (I) in precursor double-stranded RNA (dsRNA) that are extensively detected in the mammalian transcriptome ^8–10^. Epitranscriptomic adenosine-to-inosine (A-to-I) RNA editing events are widespread in the cancer transcriptome and are critical for the transition from pre-leukemic cells to fully functional LICs ^7,11–13^.

A-to-I RNA editing has a wide range of effects on RNA biology including gene expression, splicing, RNA degradation and translation, and miRNA biogenesis and/or 3’ UTR targeting ^11,14–17^. The best documented functional roles of ADAR1 are suppression of the interferon (IFN) response ^18,19^ and RNA editing of self-dsRNA to prevent abnormal activation of cytosolic self-dsRNA sensing ^17,20^. Concurrent deletion of the cytosolic dsRNA sensors melanoma differentiation-associated protein 5 (MDA5) and protein kinase R (PKR) is able to completely rescue embryo death and reverse the IFN signatures ^21–23^. ADAR1 has two isoforms, the inflammation-induced p150 that is expressed in the cytoplasm and the constitutively expressed nuclear p110, which have diverse cellular functions ^15,24–26^. The p150 isoform is thought to be the main regulator of the MDA5 pathway and is the major contributor to LIC generation in myeloid leukemia ^15,24,25,27^. In addition, recent reports suggest that the Zα-RNA binding region specific to the ADAR1 p150 isoform is responsible for the induction of IFN-stimulated genes in hematopoietic cells ^23,28,29^,

Compared to myeloid leukemia, the role of ADAR1 in lymphoid progenitor maintenance and malignant transformation is not well understood. In this study, we applied both a three-dimensional human thymic organoid system ^30^ and a T-ALL patient-derived xenograft (PDX) model to examine the function of ADAR1 in the context of T-ALL LIC maintenance. We found that the inflammation-induced ADAR1 p150 isoform is highly expressed within the LIC compartment. A thorough comparison of the A-to-I RNA editing landscape between non-relapsed and relapsed T-ALL patient cohorts revealed hyper-editing within interferon-stimulated genes (ISG). Moreover, depletion of ADAR1 inhibits LIC self-renewal and survival through both MDA5-dependent and - independent pathways based on the intrinsic expression of MDA5. Our findings indicate that deregulated RNA editing is a critical process in the generation of LICs, which has important implications for T-ALL chemoresistance and therapeutic outcomes.

## RESULTS

### ADAR1-controlled RNA epitranscriptome in relapsed T-ALL

**Figure 1:**
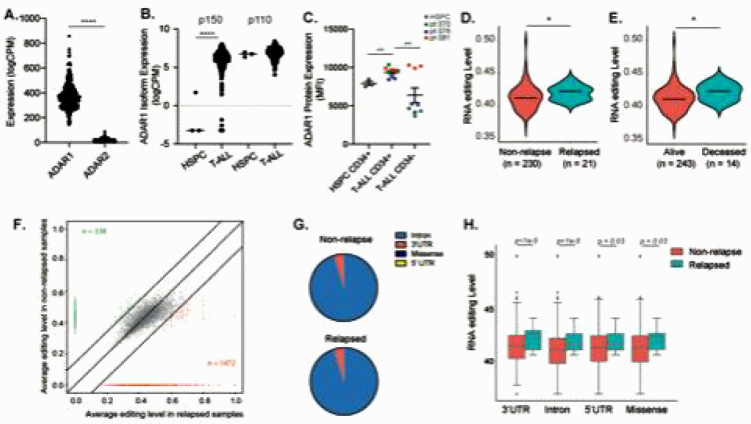
Relapsed T-ALL acquires a distinct RNA editome in contrast to non-relapsed T-ALL. **A.** Expression of *ADAR1* and *ADAR2* in T-ALL patient by RNA-seq (n = 256). **B.** Isoform expression of *ADAR1* p150 and p110 between HSPC (n=3) and T-ALL (n= 256). **C.** Quantification of ADAR1 expression in HSPCs (CD34+Lin−), T-ALL LICs (CD34+Lin−), and non-LICs (CD34−Lin+). n = 3–4 patients. **D-E.** Overall RNA editing between relapsed and non-relapsed patients (D) or between mortality status (E) in violin plots. **F.** Comparison of RNA editing level between relapsed and non-relapsed cohort display under-edited sites (green color) and overedited sites (red color) with editing levels >0.2 and detected in >10% of patients in each group. **G.** Pie chart showing RNA editing locations in non-relapsed and relapsed T-ALL. **H.** Elevated RNA editing levels across all categories of editing locations between non-relapsed and relapsed groups. Statistical analysis was calculated by unpaired student’s t-test.

Here, we investigate if RNA modifications by ADAR1 contribute to T-ALL relapse. The three isoforms within the ADAR family of RNA deaminases (ADAR1, ADAR2, and ADAR3) play different roles depending on the particular cancer type ^31^. We analyzed the expression of ADAR family genes in the NCI TARGET T-ALL dataset and discovered that the most abundant RNA editase is ADAR1 (Figures 1A). In contrast, ADAR2 is expressed at very low levels and ADAR3 expression is below detection, therefore both are unlikely to play any significant roles in T-ALL. By comparing the isoform expression between normal HSPCs and T-ALL samples, the ADAR1 p150 isoform is overexpressed in T-ALL, while the p110 expression remains constant (Figure 1B). These data were confirmed in three patient samples by intracellular flow cytometry to detect ADAR1 protein expression (Figure 1C). Interestingly, ADAR1 is expressed predominantly in the immature CD34^+^Lin^−^ population which are enriched for T-ALL LICs, instead of the more differentiated CD34^−^Lin^+^ fractions. Together these findings raise the possibility that ADAR1 may play an important role in LIC maintenance ^4,32^.

Next, we applied the A-to-I RNA editing bioinformatic pipeline to the TARGET T-ALL datasets by calculating the percentage of guanosine (G) reads at adenosine (A) at known RNA editing sites (inosines are read as guanosines) ^12^. The editing events were restricted to those detected at minimum of 10% of samples and >10 reads per site to avoid false-positives. To understand if RNA editing contributes to T-ALL relapse, the overall RNA editing levels were compared between relapsed and non-relapsed patient groups (Figures 1D–E). A significantly increased incidence of A-to-I RNA modifications is associated with both increased risk of relapse and leukemia-associated mortality. A total of 338 under-edited and 1,472 over-edited sites were found in relapsed patients compared to non-relapsed samples (Figure 1F and Table S4). Similar to previously reported work ^7,33,34^, A-to-I RNA editing occurs predominately in intronic regions, followed by 3’UTR, 5’UTR, and lastly missense or coding regions (Figure 1G). The increased editing levels were observed in all locations, indicating no selective pressure on location-specific hyper-editing during relapse (Figure 1H).

Certain molecular subtypes and early T-cell precursor (ETP) status have been associated with more aggressive disease and higher chance of developing relapse ^35,36^. We also examined if RNA editing is distinctive among different T-ALL subtypes based on the specific genomic lesions ^37–39^ (Fig. S1A). However, there was very little difference in ADAR1 expression, the overall A-to-I RNA editing levels, total number of editing sites, and editing location among various molecular subtypes. Similarly, no difference in RNA editing level is associated with sex or ETP status (Fig. S1B-C). Together, these data indicate that ADAR1 expression and elevated A-to-I RNA editing is a common attribute of relapsed T-ALL regardless of the genetic mutation status, sex, and ETP status.

### Reduction of ADAR1 impairs T-ALL LIC survival and self-renewal capacity.

**Figure 2:**
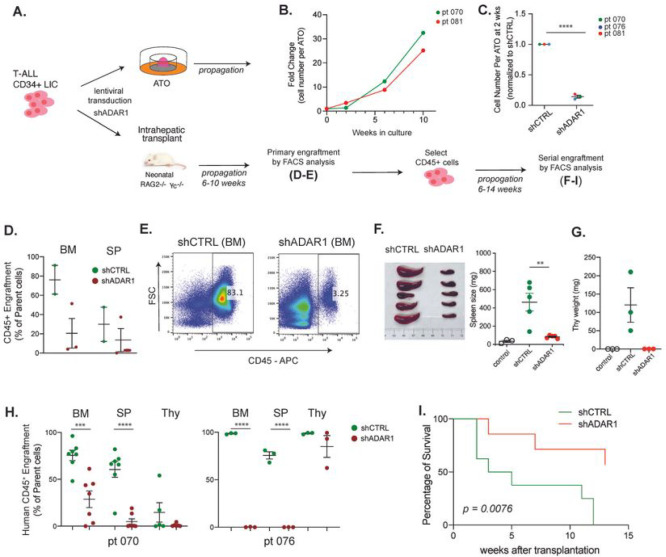
Relapsed T-ALL acquires a distinct RNA editome in contrast to non-relapsed T-ALL. **A.** Expression of *ADAR1* and *ADAR2* in T-ALL patient by RNA-seq (n = 256). **B.** Isoform expression of *ADAR1* p150 and p110 between HSPC (n=3) and T-ALL (n= 256). **C.** Quantification of ADAR1 expression in HSPCs (CD34^+^Lin^−^), T-ALL LICs (CD34+Lin^−^), and non-LICs (CD34^−^Lin^+^). n = 3–4 patients. **D-E.** Overall RNA editing between relapsed and non-relapsed patients (D) or between mortality status (E) in violin plots. **F.** Comparison of RNA editing level between relapsed and non-relapsed cohort display under-edited sites (green color) and overedited sites (red color) with editing levels >0.2 and detected in >10% of patients in each group. **G.** Pie chart showing RNA editing locations in non-relapsed and relapsed T-ALL. **H.** Elevated RNA editing levels across all categories of editing locations between non-relapsed and relapsed groups. Statistical analysis was calculated by unpaired student’s t-test.

The significant upregulation of ADAR1 and elevated A-to-I RNA editing levels in relapsed T-ALL cohorts suggest a potential role of ADAR1 in disease relapse and maintenance of LIC properties. LICs exhibit characteristics comparable to those of normal stem cells, such as self-renewal capacity, that enables them to evade chemotherapy and induce relapse ^40–42^. To examine ADAR1’s function in T-ALL LICs, we adapted the ATO system for leukemic cell expansion and established *in vivo* PDX models with high human leukemic engraftment (Figures 2 and S2A). Similar to previous reports of co-culture of T-ALL cells with MS5-DLL ^43^, the MS5-DLL4 ATO system permits successful T-ALL LIC expansion *in vitro* (Figure S2A). Primary T-ALL cells cultured in ATOs display a 2-fold expansion by week 6 and more than 20-fold by week 10 (Figure 2B–C). In PDX models, abundant human CD45^+^ leukemic engraftment is usually observed in bone marrow, spleen, and thymic hematological niches within 6–10 weeks after intrahepatic transplant into neonatal Rag2^−/−^γc^−/−^ mice (Figures 2D–H).

To study the effects of ADAR1 on self-renewal capacity in LICs, ADAR1 was knocked down by shRNA in patient-derived enriched LICs (CD34^+^Lin^−^) followed by culture in ATOs or transplantation into PDX models (Figures 2A–B). ADAR1 reduction led to decreased leukemia cell propagation (>70% reduction) in both systems (Figures 2C–E and S2B). However, the most striking effects were seen in serial transplant recipients. Equal numbers of bone marrow cells derived from lentiviral control or shADAR1 mice were transplanted to assess the self-renewal capacity of LICs. Spleen and thymus weights of mice injected with shADAR1 cells returned to the same level of non-transplanted litter controls (Figures 2F–G). In addition, ADAR1 knockdown strongly impedes serial leukemic engraftments in all hematological niches (Figure 2H). Because of the marked differences in leukemia burden between shADAR1 and control conditions, we evaluated the survival potential between these two groups. We observed significantly improved survival in shADAR1 mice (*p*<*0.0076*, Figure 2I). Together, these data suggest that ADAR1 contributes to self-renewal and survival in T-ALL LICs.

### Loss of ADAR1 reduces hyper-editing events

**Figure 3:**
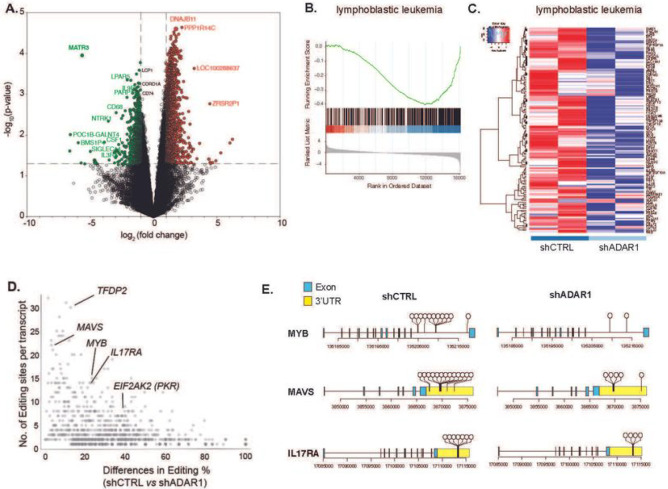
ADAR1 downregulation reduces LIC stemness gene expression. **A.** Volcano plots depicting significantly differentially expressed genes in T-ALL LICs with ADAR1 knockdown (n = 2 samples). **B-C.** Gene enrichment scores (B) and Heatmap (C) of “lymphoblastic leukemia” pathway. **D.** Analysis of differential level of RNA editing and number of A-to-I editing sites per transcript between scramble shRNA control (shCTRL) and ADAR1 knockdown. **E.** Dandelion plot of RNA editing in *MYB, MAVS* and *IL17RA*. Each dot represents a unique RNA editing event.

Since the TARGET dataset is based on bulk cell sequencing, LIC specific events could be masked. To gain better insights into LIC-specific molecular targets and pathways regulated by ADAR1, we performed RNA-seq studies on enriched T-ALL LICs (CD34^+^Lin^−^) with ADAR1 knockdown (Figures 3 and S3). Since loss of ADAR1 leads to reduced cell survival (Figure 2), the lentivirus to cell ratio was carefully titrated to obtain approximately 50% reduction of ADAR1 (Figure S5A). This allows for sufficient cell recovery after transduction for sequencing analysis. A total of 661 genes are differentially expressed upon ADAR1 knockdown, including 56 downregulated and 605 upregulated genes (Figure 3A and Table S3). A close examination of the “lymphoblastic leukemia” and the “acute undifferentiation leukemia” pathways revealed several critical self-renewal genes (e.g. *CD34, CD44, LMO2, JAK3,* and *TAL1*) were downregulated in ADAR1-deficient LICs (Figures 3B–C and S3B) ^44–46^. Interestingly, A-to-I RNA editing is rarely detected in these transcripts regardless of direction of differential expression, except for three editing sites within the *LMO2* intronic region in scramble control cells (Table S4). Similarly, the most extensively edited genes are often not differentially expressed (e.g. *IL17RA* and *EIF2AK2*), suggesting indirect regulation of ADAR1 on gene expression (Figure 3D). These data indicate that ADAR1 promotes LIC stemness by indirectly targeting self-renewal genes.

We also profiled the RNA editome landscape in FACS-sorted LICs of two T-ALL patients prior to and after ADAR1 knockdown (Figures S3C-F). Reduced ADAR1 led to a small but significant decrease in overall editing levels (Figure S3C). However, the most profound effect was the reduction in the number of editing events (~ 50%) (Figure S3D). The total number of edits decreased from 1,698 in the control to 901 in the shADAR1 condition with a predominant drop in *Alu*-enriched intronic editing sites (Figure S3E). However, the A-to-I editing level within intronic regions is not altered (Figure S3F). Since ADAR1 has a tendency to edit in clusters, a phenotype termed hyper-editing ^15,47,48^, we calculated the number of edits and changes in editing level between control and shADAR1 per each transcript. Hyper-editing is widespread in intronic (e.g. *MYB*) and 3’UTR regions of mRNA transcripts (e.g. *MAVS* and *IL17RA*) (Figures 3D–E). Therefore, ADAR1 knockdown in T-ALL LICs reduces hyper-editing events rather than the editing level at a particular site.

### T-ALL LICs pose different dependencies on dsRNA sensing by MDA5 pathway

ADAR1-directed RNA editing negatively regulates interferon (IFN) production and IFN-stimulated gene (ISG) activation by preventing accumulation of endogenous double-stranded RNA (dsRNA), which are detected by the MDA5-MAVS dsRNA sensing pathway ^17,49–51^. These roles of ADAR1 are the foundation for many of its important functions, such as preventing embryonic lethality, suppressing apoptosis during oncogenesis, and overcoming resistance to immunotherapy ^20,21,52^. However, dsRNA sensing of immunostimulatory transcripts as a mechanism in ADAR1-regulated LIC self-renewal has never been fully characterized.

**Figure 4:**
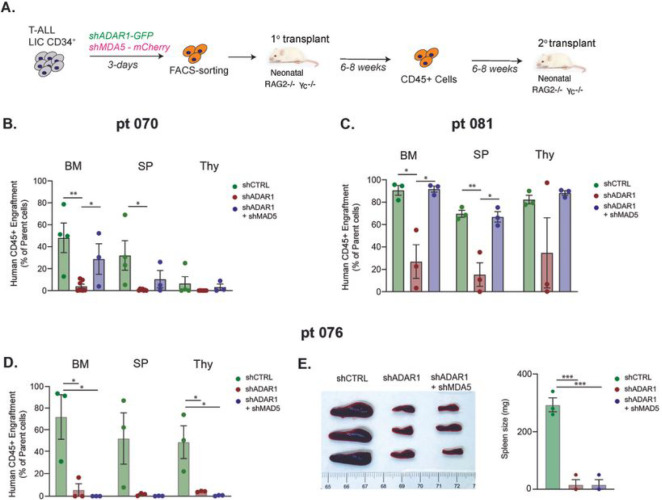
Concurrent knockdown of MDA5 and ADAR1 rescues self-renewal to various degrees in different T-ALL models. **A.** Experimental setup. T-ALL CD34^+^ cells were transduced with shCTRL, shADAR1, or shADAR1 and shMDA5 lentivirus in combination. Transduced cells were sorted based on GFP^+^mCherry^+^ (GFP for shADAR1, and mCherry for shMDA5) and serial transplant potential was measured in recipient Rag2^−/−^ gc^−/−^ mice. **B-D.** Serial leukemia engraftment and representative bone marrow FACS plot of patient 070 (**B**), patient 080 (**C**), and patient 076 (**D**) was determined for shCTRL, shADAR1, and shADAR1 in combination with shMDA5 (3–8 mice/condition). **E**. Images of spleen (left) and spleen weights (right) in serial transplanted patient 076 were determined after an 8-week engraftment interval. *p<0.05, **p<0.01, ***p<0.001, unpaired Student t-test.

To investigate this functionally, we performed concurrent knockdown of MDA5 (mCherry-labeled) and ADAR1 (EGFP-labeled) in PDX T-ALL LICs. The successfully dual-transduced cells (mCherry^+^EFGP^+^) were transplanted into immunocompromised Rag2^−/−^γc^−/−^ mice and then serial transplanted to quantify self-renewal capacity of LICs (Figure 4A). Surprisingly, co-knockdown of ADAR1 and MDA5 exhibits diverse rescue effects on self-renewal in the three PDX models tested. A partial rescue of serial leukemia engraftment was detected in co-knockdown compared to the ADAR1 deficient alone condition in PDX-070 (Figure 4B). In PDX-081, a complete rescue was observed in all hematopoietic niches (Figure 4C). In contrast, no differences in serial leukemia engraftments or spleen weight were observed in PDX-076 (Figures 4D–E). These data indicate that ADAR1-directed RNA editing controls LIC self-renewal through dsRNA sensing in at least a portion of T-ALL patients.

**Figure 5:**
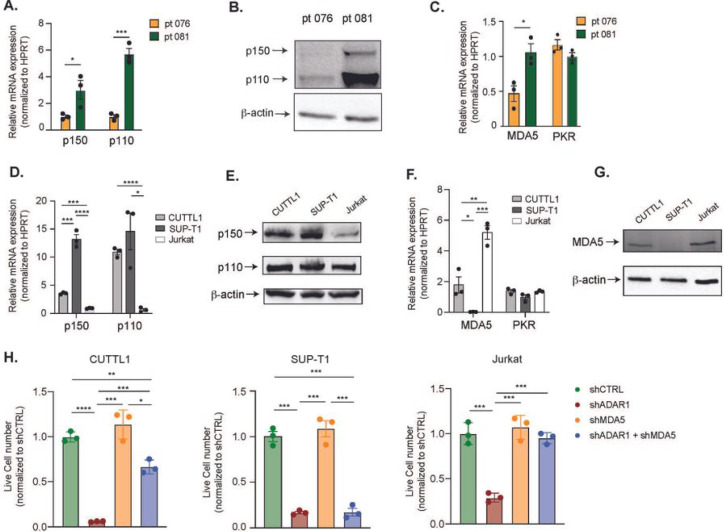
Basal expression of MDA5 controls the dependency on ADAR1-MDA5 axis. **A-B.** Expression of ADAR1 isoforms in patient 076 and patient 081 was measured by RT-qPCR and western blot in Lin^−^CD34^+^ LIC− enriched population. **C**. Expression of MDA5 was determined in Lin^−^CD34^+^ LIC-enriched cells of patient 076 and patient 081. **D-E.** Expression of ADAR1 isoforms in three T-ALL cell lines, CUTTL1, SUP-T1, and Jurkat. **F-G**. MDA5 and PKR mRNA expression (F) and protein level (G) were determined in T-ALL cell lines. **H.** Cell counts of shRNA control, shADAR1, shMDA5, and co-knockdown of shADAR1 and shMDA5 were assessed after 3-days post lentiviral transduction. Data from three independent experiments are shown. Error bars represent mean with SEM. *p<0.05, **p<0.01, ***p<0.001, and ****p<0.0001 unpaired Student t-test.

Next, we explored potential mechanisms guiding the difference in response to co-knockdown of MDA5 and ADAR1. Curiously, differential gene expressions in the Rig-I-Like signaling and cytosolic sensing pathways were detected between the PDX-070 and −076 samples, which could suggest differences in intrinsic signaling properties and dependency on dsRNA-sensing pathways among patients (Figure S4). Moreover, the ADAR1 p150 isoform is thought of as the main regulator of RNA editing in the cytoplasm and therefore is responsible for regulating dsRNA sensing by MDA5, while ADAR1 p110 is largely dispensable for MDA5 signaling^27^. Thus, the intrinsic expression of p150 and MDA5 dsRNA sensor in T-ALL patients might dictate the level of dependency on the MDA5 pathway. To test this hypothesis, we measured the expression of ADAR1 isoforms and MDA5 in FACS-enriched LICs in the three T-ALL samples (Figure 5). Patient 070 did not yield enough LICs therefore we could not complete the analysis. Patient 081 has significantly elevated expression of ADAR1 p150, p110, as well as MDA5, compared to patient 076 (Figures 5A–C). The level of another dsRNA sensor PKR was also determined and showed no difference between patients. Coupled with the differential rescue effects of MDA5 and ADAR1 co-knockdown (Figure 4C), our data support that patient 081 relies on the ADAR1 p150-MDA5 axis for promoting self-renewal, while patient 076 likely depends on both p150-MDA5 axis and p110 dependent mechanisms.

We next sought to validate whether this isoform-specific dependency of ADAR1 is also presented in T-ALL cell lines. We first evaluated the endogenous expression of the p150 and p110 isoforms, and dsRNA sensors MDA5 and PKR, in CUTTL1, SUP-T1, and Jurkat cells. SUP-T1 has the highest expression of p150 isoform, followed by CUTTL1 and then Jurkat cells (Figures 5D–E). The protein expression of p110 is comparable among the three cell lines as shown in western blot analysis (Figure 5E). MDA5 is expressed at the highest level in Jurkat and lowly expressed in CUTTL1 and SUP-T1 (Figure 5F–G). Consistent with T-ALL patient LICs, the expression of PKR does not vary significantly among the cell lines (Figure 5F). Next, we performed ADAR1 knockdown alone, MDA5 knockdown alone, or MDA5 and ADAR1 co-knockdown to examine if partial or complete rescue effects are presented (Figures S5A-B). Successfully transduced cells were FACS-sorted based on shMDA5 (mCherry^+^) and shADAR1 (EGFP^+^) signals and cell propagation and apoptosis rate were evaluated (Figures 5H and S5C-D). Knockdown of MDA5 alone does not significantly alter cell proliferation (Figure 5H). Silencing of ADAR1 reduced cell proliferation and induced apoptosis in all cell lines, suggesting a universal dependency on RNA editing in T-ALL (Figures 5H and S5C-D). Interestingly, similar to T-ALL PDX models, co-knockdown of ADAR1 and MDA5 completely rescued these effects of ADAR1 knockdown in Jurkat, while only partial rescue was observed in CUTTL1. Consistent with the low MDA5 expression, no significant difference was observed in shADAR1 compared with co-knockdown condition in SUP-T1 cells. Thus, the phenotypic differences in p150-MDA5 dependency predominately reflect the level of MDA5 expression, as opposed to p150 level in T-ALL models.

### ADAR1 RNA editing-independent activity promotes nuclear localization of dsRNA

**Figure 6:**
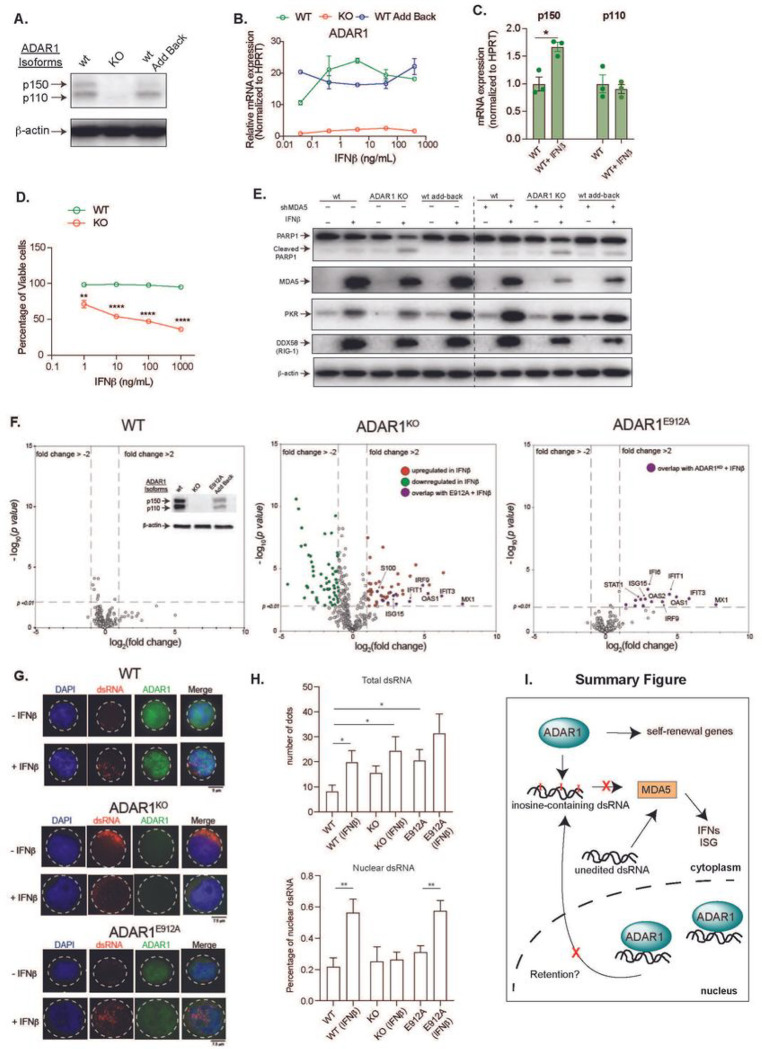
RNA editing-dependent and -independent mechanisms of ADAR1 suppress aberrant dsRNA sensing. **A.** Western blot showing ADAR1 expression in modified Jurkat T-ALL cell lines, including wildtype (wt), ADAR1 KO (KO), and added back wildtype ADAR1 by lentiviral overexpressing vector (add back). b-actin was used as loading control. **B**. Jurkat cells were stimulated with various concentrations of IFNb and the gene expression of ADAR1 was determined (n = 2 independent experiments). **C**. Quantification of ADAR1 p150 and p110 isoforms upon IFNb treatment at 10 ng/mL for 24 hours (n = 3 independent experiments). **D**. Cell viability was quantified in Jurkat cells upon IFNb stimulation (n = 3 independent experiments). **E**. Western blot showing levels of dsRNA sensor (PRK, MDA5, DDX58) and apoptosis markers (PARP1 and cleaved PARP1). Cells were stimulated with IFNb (1 ng/mL) for 48 hours. b-actin is used as loading control. **F**. NanoString analysis of gene expression in wildtype (wt), ADAR1 KO, and ADAR1^E912A^ overexpressing Jurkat cells stimulated with IFNb (1ng/mL, 48 hrs), (n = 2 independent experiments). Insert: western blots showing ADAR1 expression in wildtype, ADAR1 KO, and ADAR1^E912A^ add back cells. **G,**Immunofluorescent staining to detect the localization of dsRNA (J2 antibody) in wildtype (wt), ADAR1 KO, and ADAR1^E912A^ Jurkat cells stimulated with IFNb (1 ng/mL, 24 hrs). **H**. Quantification of total dsRNA dots and percentage of nuclear dsRNA from Jurkat cells treated with IFNb (1 ng/mL, 24 hrs, 10 cells/condition. **I**. Model of pathways mediated by ADAR1 in T-ALL LICs. *p<0.05, **p<0.01, and ****p<0.0001, unpaired student t-test. Error bars represent mean with SEM in all graphs. Error bars represent mean with SEM in all graphs.

ADAR1 can operate as a dsRNA binding protein with functions independent of editing activity to promote cancer progression ^15,34,53,54^. To explore RNA editing dependent and independent mechanisms of ADAR1, we first generated an ADAR1 knockout Jurkat T-ALL cell line and a wildtype “add-back” cell line by re-expressing wildtype ADAR1 p150 isoform (ADAR1^WT^) in knockout cells with a lentiviral overexpression vector (Figure 6A). The p150 mRNA expressed both p150 and p110 isoforms due to leaky ribosome scanning ^26^. To activate the IFN response, these cells were treated with various doses of IFNα, β, and γ for 48 hours to examine changes in cell viability and ADAR1 expression (Figures 6B and S6A). IFN treatment upregulates ADAR1 in wildtype and ADAR1^WT^ add-back cells, while knockout cells had little response. As expected, exposure to IFNβ predominately upregulates the p150 isoform rather than p110 (Figure 6C). Moreover, loss of ADAR1 induces apoptosis upon IFNβ treatment as demonstrated by elevated levels of cleaved PARP1 and a reduced percentage of viable cells (Figures 6D–E).

In addition to MDA5-MAVS signaling, ADAR1 also suppresses cytoplasmic dsRNA sensing through RIG-I (retinoic acid-inducible gene I) and PKR pathways ^17,23,55,56^. While ADAR1 KO or add-back do not change MDA5, PKR, and RIG-I levels, IFNβ stimulated the expression of these dsRNA sensors in wildtype, ADAR1 KO, and ADAR1^WT^ cells, (Figure 6E). This was confirmed by elevated *PKR* expression upon addition of IFNα and IFNγ (Figure S6B). Interestingly, knockdown of *MDA5* in *ADAR1* knockout cells abrogated the IFNβ-induced RIG-I activation, but increased PKR activation, suggesting MDA5 may crosstalk with other dsRNA sensors in the combination of ADAR1 loss and IFNβ treatment (Figure 6E).

Next, we introduced a catalytically inactive mutant of the ADAR1 p150 isoform (ADAR1^E912A^) ^15,18,50^ in ADAR1 KO cells (Figure 6F). The lentiviral construct produces both p150 and p110 isoforms as previously reported ^26^. The expression of a selected panel of 790 ISGs using the Nanostring nCounter System was quantified in IFNβ stimulated wildtype, ADAR1 KO, and ADAR1^E912A^ cells. The wildtype cells are relatively insensitive to IFNβ treatment compared to ADAR1 KO cells. A total of 27 differentially expressed ISGs in ADAR1^E912A^ cells in comparison to 237 ISGs in ADAR1 knockout and 30 ISGs in wildtype cells was found (Table S5). Surprisingly, ADAR1^E912A^ overexpression was able to rescue all downregulated targets found in the IFNβ treated KO condition as well as suppress the majority of upregulated ISGs. Approximately 70% (19 out of 27) of ADAR1^E912A^ regulated targets overlapped with those of ADAR1 knockout cells, while only 3.7% (1 out of 27) of targets overlapped with IFN-treated wildtype cells. Together with the cell lethality and functional rescue by MDA5 depletion, these data suggest that ADAR1’s editing-independent function also contributes to suppress MDA5 signaling in T-ALL.

The activation of dsRNA sensing pathways depends on cellular localization and length of the accessible endogenous dsRNA ^57^. The inosine containing dsRNA can be retained in the nucleus by p54^nrb^, PSF, and MATR3, thus avoiding export to the cytoplasm and detection by MDA5 ^58^. Therefore, we hypothesized that ADAR1^E912A^ may retain dsRNA in the nucleus to limit the cytosolic dsRNA pool. Immunofluorescent staining using a J2 dsRNA antibody was applied to identify the cellular dsRNA localization prior to and after IFNβ treatment (Figures 6G–H). The IFNβ treatment length was reduced to 24 hours to detect dsRNA localization prior to cell death. Wildtype cells respond to the addition of IFNβ by increasing total dsRNA level (2.4 folds) and the percentage of nuclear dsRNA (average 56%). The same nuclear retention phenotype was also observed in ADAR1^E912A^ cells (average 57%). Interestingly, in the absence of IFNβ, both ADAR1 knockout and ADAR1^E912A^ cells showed elevated total dsRNA levels compared to wildtype cells without triggering cell death, suggesting a certain level of unedited dsRNA is tolerated. However, the most striking effect was seen in IFNβ treated knockout cells. We detected an abundant quantity of dsRNA accumulated in the cytoplasm. However, the level of nuclear dsRNA remained constant prior to and after IFNβ treatment. To confirm this finding, we exposed CUTTL1 cell line to IFNβ for 24 hours and analyzed dsRNA location (Figure SC-D). While the total dsRNA level did not change, the nuclear dsRNA showed a significant increase from 35% to 62%, indicating this retention mechanism is commonly used by T-ALL cells in response to IFN stress. Together, these data reveal an important RNA editing-independent mechanism of ADAR1 in preventing MDA5-directed dsRNA sensing.

## DISCUSSION

T-ALL is an aggressive hematological malignancy which arises from transformation of lymphoid progenitors with the cooperation of tumor suppressors and oncogenes ^59–61^. We now understand that RNA modifications such as A-to-I RNA editing and m6A are critical in promoting cancer progression and therapeutic resistance ^7,62^. We have shown RNA editing by ADAR1 is an important regulatory mechanism required for HSC maintenance and transformation into myeloid leukemia ^15,20,24,34,63^. However, the role of ADAR1 in lymphoid neoplasms such as T-ALL has never been explored. Here, we described a fundamental role of ADAR1 in maintenance of T-ALL LICs. Increased RNA editing and ADAR1 expression was detected in relapsed T-ALL patients, regardless of the molecular genetic mutations. ADAR1 directs A-to-I hyper-editing of ISG dsRNA and retains unedited nuclear dsRNA to avoid detection by the innate immune sensor MDA5. Consequently, loss of ADAR1 leads to impaired survival and self-renewal and improves overall survival in PDX models. Finally, we demonstrated that ADAR1 regulates LIC self-renewal through aberrant dsRNA sensing in a portion of T-ALL samples depending on the expression level of the dsRNA sensor MDA5.

We have previously discovered ADAR1’s contribution to neoplastic transformation of myeloid LICs via several different pathways: 1) regulation of self-renewing microRNA biogenesis, 2) editing of 3’UTR of oncogenes to prevent miRNA-directed degradation, 3) editing of coding genes, and 4) induction of oncogenic RNA splicing events ^15,24,25,64^. Whether editing of immunogenic dsRNA and suppression of aberrant dsRNA sensing could enhance LIC self-renewal capacities is an important question that has not been extensively addressed. We provide the link between malignant A-to-I RNA editing and suppression of dsRNA sensing as a mechanism in promoting LIC self-renewal. Hyper-editing events are commonly observed in ISG genes within intronic and 3’UTR regions. We identified unexpected differences among three T-ALL models in response to concurrent knockdown of MAD5 and ADAR1. This novel phenomenon could stem from the intrinsic tolerance of unedited dsRNA or variable levels of dsRNA sensors among patients. We further reported that the intrinsic expression of MDA5, rather than p150 or p110 isoforms, dictates the level of response to co-knockdown. Since the ADAR1 p150 isoform specifically regulates the MDA5-MAVS pathway^27^, our study suggests that LIC self-renewal and survival rely entirely on the p150-MDA5 axis and the p110 isoform is likely dispensable in such scenarios. In contrast, other T-ALL models may depend on both p150 and p110 isoforms in a MDA5-independent manner. It is possible that LICs possess different levels of dependency on ADAR1 due to the diverse ISG signatures in T-ALL patients as previously reported^50^. Future studies are necessary to definitively decouple the isoform-specific function, RNA editing targets, and pathways regulated by a particular isoform in a large cohort of patients and potentially other tumor types.

Lastly, we report an RNA editing-independent role of ADAR1 in attenuating aberrant dsRNA sensing. To trigger dsRNA sensing by cytosolic MDA5, unedited endogenous dsRNA in the absence of ADAR1 must be in the cytoplasm. The deaminase deficient ADAR1^E912A^ was able to suppress the majority of ISGs in ADAR1 knockout cells, in part through nuclear dsRNA retention to limit the cytosolic dsRNA pool. However, we cannot rule out the possibility that ADAR1^E912A^ competes with MDA5 to prevent efficient dsRNA sensing. Surprisingly, we noticed that T-ALL cells can tolerate a certain level of unedited cytosolic dsRNA without triggering apoptosis. It is curious as to how cancer cells set this limit using the complex and diverse regulatory dsRNA sensing network, which may contribute to the difference in response to cytosolic dsRNA in T-ALL models. In conclusion, this work highlights the intrinsic differences in how ADAR1 regulates malignant dsRNA sensing and promotes self-renewal among patient samples, in addition to mechanistic details of LIC maintenance. This in turn opens the door for therapeutic targeting of these downstream processes to prevent relapse and therapeutic resistance.

## ONLINE METHODS

### CONTACT for REAGENT AND RESOURCE SHARING

Further information and requests for resources and reagents should be directed to and will be fulfilled by the Lead Contact, Qingfei Jiang (q1jiang@health.ucsd.edu).

### EXPERIMENTAL MODEL AND SUBJECT DETAILS

#### Animal Experiments

All mouse studies were conducted under protocols approved by the Institutional Animal Care and Use Committee (IACUC) of the University of California, San Diego and were in compliance with federal regulations regarding the care and use of laboratory animals: Public Law 99–158, the Health Research Extension Act, and Public Law 99–198, the Animal Welfare Act which is regulated by USDA, APHIS, CFR, Title 9, Parts 1, 2, and 3. Immunocompromised Rag2^−/−^γc^−/−^ mice were bred and maintained in the Sanford Consortium for Regenerative Medicine vivarium according to IACUC approved protocols of the University of California, San Diego. Neonatal mice of both sexes were used in the study. T-ALL CD34^+^ or CD45^+^ cells were injected intrahepatically into 2–3 days oldneonatal Rag2^−/−^γc^−/−^ mice. Leukemic engraftment was quantified by FACS analysis-based peripheral blood screening of human CD45^+^ population starting from week 6 for every 2 weeks until the engraftment exceeded 1%. Mice were then humanely sacrificed, and cells were collected from hematological organs (bone marrow, spleen and thymus) for FACS analysis.

#### Human Subjects

Patient T-ALL samples were obtained from consenting patients at the University of California, San Diego in accordance with an approved human research protections program Institutional Review Board approved protocol (#130794) that meets the requirements as stated in 45 CFR 46.404 and 21 CFR 50.51. De-identified (IRB exempt) human cord blood samples were purchased as purified CD34^+^ cells from AllCells Inc or StemCell Techologies Inc. Detailed patient information can be found in Table S2.

### METHOD DETAILS

#### Patient sample preparation.

Peripheral blood mononuclear cells (PBMC) were extracted by Ficoll density centrifugation. CD45^+^ or CD34^+^ cells were purified using magnetic columns (MACS, Miltenyi) or FACS sorted with human-specific antibody according to published methods in FACSAria II ^15^.

#### Cell culture

CUTTL1, SUP-T1 and Jurkat human cell lines were cultured in 37°C in DMEM supplemented with 10% FBS and 2 mM L-glutamine and maintained according to ATCC protocols. MS5-DLL1 and MS5-DLL4 were maintained in high glucose DMEM with 10% FBS and 1X penicillin-streptomycin according to previous protocol ^30,65^. All cell lines were confirmed to be mycoplasma-free with repeated testing and authenticated by short-tandem repeat (STR) profiling. ADAR1 knockout cell line was generated by Sythego. Wildtype ADAR1 and mutant ADAR1^E912A^ were introduced into the knockout Jurkat cells by transduction of wild-type and mutant ADAR1^E912A^ lentivirus. Stable ADAR1 expression were confirmed by RT-qPCR and western blot every 5 passages.

#### Lentiviral construct and overexpression.

Lentiviral vectors (pLV-shRNA-EGFP or mCherry:T2A:Puro-U6) was purchased (VectorBuilder) and wild-type and mutant ADAR1^E912A^ (pCDH-EF1-T2A-copGFP) were produced according to published protocol ^25^. All lentivirus was titer by transduction of HEK293T cells and efficiency was assessed by p24 ELISA and RT-qPCR of the 5’ LTR region. Lentiviral transduction of primary T-ALL or cord blood samples was performed at a MOI of 100–200. The cells were cultured for 3–4 days in 96-well plate (2X10^5^-5X10^5^ cells per well) containing StemPro (Life Technologies) media supplemented with human IL-6, stem cell factor (SCF), Thrombopoietin (TPO) and FLT3 (all from R&D Systems). For T-ALL cell lines, the cells were transduced at a MOI of 20–50 in culture media.

#### ATO 3D organoid culture.

ATO organoid experiments were performed as previously described ^30^. The MS5 mouse stromal cells were engineered to co-express human DLL1 or DLL4 NOTCH ligand and EGFP marker. MS5-DLL1 and -DLL4 were cultured up to 20 passages and the cells will be authenticated every 5 passages by flow of EGFP signal and examining the DLL1 or DLL4 expression by RT-qPCR. To generate ATOs, CD34^+^ cells (2–5×10^3^ cells per ATO) were isolated from T-ALL patients by magnetic selection (Miltenyibiotech) and combined with MS5-DLL1/DLL4 cells at 1:20 ratio, seeded on a 0.4 μm Millicell transwell insert (EMD Millipore), and placed into 6-well plate with serum-free culture media supplemented by recombinant IL7 (50 ng/mL) and FLT3 (50 ng/mL). ATOs were cultured up to 20 weeks. The cells were harvested by adding staining media (ice-cold PBS with 2% FBS and 2 mM EDTA) to each well and pipetting to dissociate ATOs. Cells were then immunostained with antibodies (Table S1) and analyzed on a BD Aria Fusion and with FlowJo.

#### Patient derived xenograft transplantation

To establish T-ALL models, freshly ficolled cells were transplanted intrahepatically into neonatal Rag2^−/−^γc^−/−^ mice (5×10^5^ – 1×10^6^ per pup) according to our preciously published methods ^15,25^. Bone marrow, spleen and thymus tissues were harvested after 6–20 weeks and stored in liquid nitrogen. For functional studies, CD34^+^ or CD45^+^ cells were transduced with lentiviral vectors for 2–3 days. Cells were harvested in staining media, counted, and equal numbers of cells per condition were transplanted into recipient mice (5×10^4^ – 1×10^5^ per pup). Transplanted mice were FACS screened for human engraftment in peripheral blood at 6–10 weeks. Once human engraftment was confirmed (>1% human CD45^+^ cells in peripheral blood), mice were euthanized, and single cell suspensions of hematopoietic tissues (bone marrow, spleen, and thymus) were analyzed by FACS and FlowJo.

#### Flow cytometry analysis and sorting

Flow staining was performed in staining media for 30 min on ice in the dark. Cells were blocked using FcR block (Biolegend, San Diego, CA) for 15 minutes before antibody staining with to a final dilution of 1:25. DAPI solution was added before analysis to exclude dead cell debris. Analysis and sorting was performed on BD Aria Fusion, Aria II or Fortessa. Sorted cells were collected into staining media filled FACS tubes or 1.7mL Eppendorf tubes. The LICs are evaluated by the corresponding cell surface markers (Table S1). For intracellular ADAR1 staining, cells were stained with ethidium monoazide (EMA) for 15 min in the dark and then 15 min under light, followed by cell surface staining. After washing in staining buffer, cells were fixed and permeabilizated with an intracellular buffer set (eBioscience, San Diego, CA) and intracellularly stained with an antibody against ADAR1 (Abcam, ab126745) at 1:100 dilution. Secondary antibody of Alexa488 or Alexa647 were used to amplify ADAR1 signals.

#### RNA extraction and quantitative real-time polymerase chain reaction.

Total RNA was isolated using RNeasy Micro kit or Mini kit (Qiagen) and the quality was determined by NanoDrop. Complementary DNA was synthesized according to published methods ^15^. qRT-PCR was performed in duplicate or triplicate on an CFX384 with the use of SYBR GreenER qPCR SuperMix (Invitrogen), 5 ng of template cDNA and 0.2 μM of each forward and reverse primer. Human specific HPRT primers were used as housekeeping control. Quantitative values were obtained from the cycle number (Cq value) using the Bio-Rad Maestro Software. The RT-qPCR primers are shown in Table S6.

#### Western blots

Cell lysate (10 μg) was mobilized onto a nitrocellulose membrane after electrophoresis on a 10% SDS- acrylamide gel. The membrane was blocked in 5% BSA/20 mM Tris-HCl for 30 min. The blot was incubated with primary antibody in 5% BSA/20 mM Tris-HCl/0.1% Tween-20 overnight at 4°C, followed by secondary HPR-linked Rabbit IgG antibody (Cell Signaling, #70745) for 2 hr at room temperature. Membrane was then incubated in SuperSignal West Femto Substrate (ThermoFisher, #34096) for chemiluminescent reading on ChemiDoc System (Bio-Rad).

#### Interferon stimulation assay

Cells were seeded at a density of 10^5^ in a 12-well plate and treated with a single dose of IFNα, IFNβ, or IFNγ (R&D Systems) at 0.05–500 ng/mL. After 48 hours, cells were harvested and analyzed by western blot and RT-qPCR. The cell supernatants were collected and the S100A9 level was determined by an ELISA assay (Invitrogen). Cell viability was determined trypin blue assay.

#### Nanostring nCounter

Jurkat cells were collected after 24 hours of IFNβ-stimulation and RNA was isolated (RNeasy Plus mini kit, Qiagen). The mRNA levels were directly measured using the Human CAR-T characterization panel kit with additional custom probes (Table S5) from NanoString nCounter gene expression system (NanoString). The differential expression analyses of mRNA were performed using nSolver software (NanoString) and visualized in Prism software.

#### Immunofluorescence staining

Cells (1–2×10^3^) were harvested in ice-cold PBS and loaded on adhesion slides (Marienfeld Superior) by incubating for 10 min at room temperature. The slides were transferred into a coplin jar containing ice-cold PBS for 5 min and fixed with 4% paraformaldehyde in PBS for 10 min at room temperature. Immunofluorescence was performed by immersing slides in PBST (1x PBS with 0.05% Tween-20). Slides were overlaid with blocking solution (2% fetal bovine serum in PBST) for 1 hour at room temperature. After washing, primary antibody was added to the slides and incubated overnight at 4C. Secondary antibody was overlaid to spotted cells for 1 hour in the dark. DAPI was added and the slides were sealed with a coverslip. Imaging was performed using a Keyence confocal microscope. The intensity and numbers of dsRNA foci were caculated using ImageJ software.

#### Whole RNA-sequencing

Samples with RNA integrity numbers (RIN) ≥7 will be processed using SMART cDNA synthesis and NEBNext paired-end DNA Sample Prep Kit to prepare libraries. RNA-sequencing were performed on NovaSeq 6000 S4 with 150bp paired-end reads. T-ALL RNA sequencing dataset were obtained from data generated by the Therapeutically Applicable Research to Generate Effective Treatments (https://ocg.cancer.gov/programs/target) initiative, phs000464. The data used for this analysis are available at https://portal.gdc.cancer.gov/projects.” The minimal reads per sample was 50M to ensure optimal RNA editing calling.

#### Transcript and gene quantification and differential expression

Reads were aligned using STAR’s two-pass alignment method, using the GRCh38.84 reference genome and corresponding Ensembl GTF ^66,67^. STAR was used to output a sorted genome-coordinate based BAM file, as well as a transcriptome-coordinate based BAM file ^68^. STAR also was used to output the number of reads aligned to each gene for gene expression quantification. STAR settings were based on those used for the ENCODE STAR-RSEM pipeline. The R BioConductor packages EdgeR ^69^ and limma ^70^ were used to implement the limma-voom method for differential expression analysis. Low expressed genes with counts per million (cpm) < 1 in at least 1 of the samples were filtered out and then trimmed mean of M-values (TMM) ^71^ normalization was applied. The infer_experiment.py script from the RSeQC package was used to confirm the strandedness option corresponding to the correct read counts ^72,73^ and to confirm the forward strand probability for input to RSEM. The total reads per million (TPM) ^74^ over the total collapsed exonic regions represent the ‘gene’ expression level. Significant differentially expressed genes (p < 0.05) with log fold value for each comparison was used to perform Gene Set Enrichment Analysis. The R Bioconductor packages DOSE^1^ and clusterProfile^2^ was used to implement GSEA and visualize the results based on MSigDb and Disease ontology database. Heatmaps visualize the log_2_(TPM+1) transformed TPM quantity from RSEM for each feature and were generated using GENE-E with default settings for a row and column clustered heatmap and dendrogram.

#### RNA editing analysis

Coordinates from the DARNED and RADAR databases were combined and converted to GRCh38 using Crossmap ^75–77^. The resulting coordinates were used as input to the REDItoolKnown.py script from the REDItools package to determine the number of A, C, G, and T base calls at each coordinate ^78^. Only coordinates with coverage greater than or equal to 5 in all samples for a given comparison were reported. The percentage of bases called as G at bases with reference A was reported. Coordinates with a percentage G of 0 in all samples for a given sample were not reported. Using percentage G at a coordinate as an input metric, the mean percentage G in each group, the log_2_ fold change of percentage G of one group versus another, the p values, and minus log_10_ p values by both the Wilcox and student t-tests were recorded for each coordinate similar to published methods ^24^. Coordinates were annotated with the name of the closest gene using bedtools closest and bedtools intersect ^79^.

#### Other Statistical Analysis and Reproducibility

All experiments were performed with at least two biological or experimental replicates, with specific number of replicates stated in the figure legends. Unless otherwise stated, the statistical analyses were performed using GraphPad Prism (v7.0) and statistical significance were determined at p value < 0.05, with specific statistical test stated in the figure legends.

## References

[1] Karrman K., and Johansson B. (2017). Pediatric T-cell acute lymphoblastic leukemia. Genes Chromosomes Cancer 56, 89–116. 10.1002/gcc.22416. 27636224

[2] Hefazi M., and Litzow M.R. (2018). Recent Advances in the Biology and Treatment of T Cell Acute Lymphoblastic Leukemia. Curr Hematol Malig Rep 13, 265–274. 10.1007/s11899-018-0455-9. 29948644

[3] McMahon C.M., and Luger S.M. (2019). Relapsed T Cell ALL: Current Approaches and New Directions. Curr Hematol Malig Rep 14, 83–93. 10.1007/s11899-019-00501-3. 30880359

[4] Ma W., Gutierrez A., Goff D.J., Geron I., Sadarangani A., Jamieson C.A., Court A.C., Shih A.Y., Jiang Q., Wu C.C., (2012). NOTCH1 signaling promotes human T-cell acute lymphoblastic leukemia initiating cell regeneration in supportive niches. PLoS One 7, e39725. 10.1371/journal.pone.0039725. 22768113 PMC3387267

[5] Vicente C., and Cools J. (2015). The origin of relapse in pediatric T-cell acute lymphoblastic leukemia. Haematologica 100, 1373–1375. 10.3324/haematol.2015.136077. 26521295 PMC4825310

[6] Goossens S., and Van Vlierberghe P. (2014). Controlling pre-leukemic thymocyte self-renewal. PLoS Genet 10, e1004881. 10.1371/journal.pgen.1004881. 25522333 PMC4270492

[7] Jiang Q., Crews L.A., Holm F., and Jamieson C.H.M. (2017). RNA editing-dependent epitranscriptome diversity in cancer stem cells. Nat Rev Cancer 17, 381–392. 10.1038/nrc.2017.23. 28416802 PMC5665169

[8] Paul M.S., and Bass B.L. (1998). Inosine exists in mRNA at tissue-specific levels and is most abundant in brain mRNA. EMBO J 17, 1120–1127. 10.1093/emboj/17.4.1120. 9463389 PMC1170460

[9] Levanon E.Y., Eisenberg E., Yelin R., Nemzer S., Hallegger M., Shemesh R., Fligelman Z.Y., Shoshan A., Pollock S.R., Sztybel D., (2004). Systematic identification of abundant A-to-I editing sites in the human transcriptome. Nat Biotechnol 22, 1001–1005. 10.1038/nbt996. 15258596

[10] Polson A.G., Crain P.F., Pomerantz S.C., McCloskey J.A., and Bass B.L. (1991). The mechanism of adenosine to inosine conversion by the double-stranded RNA unwinding/modifying activity: a high-performance liquid chromatography-mass spectrometry analysis. Biochemistry 30, 11507–11514. 10.1021/bi00113a004. 1747369

[11] Eisenberg E., and Levanon E.Y. (2018). A-to-I RNA editing - immune protector and transcriptome diversifier. Nat Rev Genet 19, 473–490. 10.1038/s41576-018-0006-1. 29692414

[12] Jiang Q., Isquith J., Ladel L., Mark A., Holm F., Mason C., He Y., Mondala P., Oliver I., Pham J., (2021). Inflammation-driven deaminase deregulation fuels human pre-leukemia stem cell evolution. Cell Rep 34, 108670. 10.1016/j.celrep.2020.108670. 33503434 PMC8477897

[13] Han L., Diao L., Yu S., Xu X., Li J., Zhang R., Yang Y., Werner H.M., Eterovic A.K., Yuan Y., (2015). The Genomic Landscape and Clinical Relevance of A-to-I RNA Editing in Human Cancers. Cancer Cell 28, 515–528. 10.1016/j.ccell.2015.08.013. 26439496 PMC4605878

[14] Nishikura K. (2016). A-to-I editing of coding and non-coding RNAs by ADARs. Nat Rev Mol Cell Biol 17, 83–96. 10.1038/nrm.2015.4. 26648264 PMC4824625

[15] Jiang Q., Isquith J., Zipeto M.A., Diep R.H., Pham J., Delos Santos N., Reynoso E., Chau J., Leu H., Lazzari E., (2019). Hyper-Editing of Cell-Cycle Regulatory and Tumor Suppressor RNA Promotes Malignant Progenitor Propagation. Cancer Cell 35, 81–94 e87. 10.1016/j.ccell.2018.11.017. 30612940 PMC6333511

[16] Wang I.X., So E., Devlin J.L., Zhao Y., Wu M., and Cheung V.G. (2013). ADAR regulates RNA editing, transcript stability, and gene expression. Cell Rep 5, 849–860. 10.1016/j.celrep.2013.10.002. 24183664 PMC3935819

[17] Chung H., Calis J.J.A., Wu X., Sun T., Yu Y., Sarbanes S.L., Dao Thi V.L., Shilvock A.R., Hoffmann H.H., Rosenberg B.R., and Rice C.M. (2018). Human ADAR1 Prevents Endogenous RNA from Triggering Translational Shutdown. Cell 172, 811–824 e814. 10.1016/j.cell.2017.12.038. 29395325 PMC5831367

[18] Rice G.I., Kasher P.R., Forte G.M., Mannion N.M., Greenwood S.M., Szynkiewicz M., Dickerson J.E., Bhaskar S.S., Zampini M., Briggs T.A., (2012). Mutations in ADAR1 cause Aicardi-Goutieres syndrome associated with a type I interferon signature. Nat Genet 44, 1243–1248. 10.1038/ng.2414. 23001123 PMC4154508

[19] Hartner J.C., Walkley C.R., Lu J., and Orkin S.H. (2009). ADAR1 is essential for the maintenance of hematopoiesis and suppression of interferon signaling. Nat Immunol 10, 109–115. 10.1038/ni.1680. 19060901 PMC2701568

[20] Liddicoat B.J., Piskol R., Chalk A.M., Ramaswami G., Higuchi M., Hartner J.C., Li J.B., Seeburg P.H., and Walkley C.R. (2015). RNA editing by ADAR1 prevents MDA5 sensing of endogenous dsRNA as nonself. Science 349, 1115–1120. 10.1126/science.aac7049. 26275108 PMC5444807

[21] Mannion N.M., Greenwood S.M., Young R., Cox S., Brindle J., Read D., Nellaker C., Vesely C., Ponting C.P., McLaughlin P.J., (2014). The RNA-editing enzyme ADAR1 controls innate immune responses to RNA. Cell Rep 9, 1482–1494. 10.1016/j.celrep.2014.10.041. 25456137 PMC4542304

[22] George C.X., Ramaswami G., Li J.B., and Samuel C.E. (2016). Editing of Cellular Self-RNAs by Adenosine Deaminase ADAR1 Suppresses Innate Immune Stress Responses. J Biol Chem 291, 6158–6168. 10.1074/jbc.M115.709014. 26817845 PMC4813567

[23] Maurano M., Snyder J.M., Connelly C., Henao-Mejia J., Sidrauski C., and Stetson D.B. (2021). Protein kinase R and the integrated stress response drive immunopathology caused by mutations in the RNA deaminase ADAR1. Immunity 54, 1948–1960 e1945. 10.1016/j.immuni.2021.07.001. 34343497 PMC8446335

[24] Jiang Q., Crews L.A., and Jamieson C.H. (2013). ADAR1 promotes malignant progenitor reprogramming in chronic myeloid leukemia. Proceedings of the National Academy of Sciences of the United States of America 110, 1041–1046. 10.1073/pnas.1213021110. 23275297 PMC3549099

[25] Zipeto M.A., Court A.C., Sadarangani A., Delos Santos N.P., Balaian L., Chun H.J., Pineda G., Morris S.R., Mason C.N., Geron I., (2016). ADAR1 Activation Drives Leukemia Stem Cell Self-Renewal by Impairing Let-7 Biogenesis. Cell Stem Cell. 10.1016/j.stem.2016.05.004. PMC4975616 27292188

[26] Sun T., Yu Y., Wu X., Acevedo A., Luo J.D., Wang J., Schneider W.M., Hurwitz B., Rosenberg B.R., Chung H., and Rice C.M. (2021). Decoupling expression and editing preferences of ADAR1 p150 and p110 isoforms. Proceedings of the National Academy of Sciences of the United States of America 118. 10.1073/pnas.2021757118. PMC8000508 33723056

[27] Pestal K., Funk C.C., Snyder J.M., Price N.D., Treuting P.M., and Stetson D.B. (2015). Isoforms of RNA-Editing Enzyme ADAR1 Independently Control Nucleic Acid Sensor MDA5-Driven Autoimmunity and Multi-organ Development. Immunity 43, 933–944. 10.1016/j.immuni.2015.11.001. 26588779 PMC4654992

[28] de Reuver R., Dierick E., Wiernicki B., Staes K., Seys L., De Meester E., Muyldermans T., Botzki A., Lambrecht B.N., Van Nieuwerburgh F., (2021). ADAR1 interaction with Z-RNA promotes editing of endogenous double-stranded RNA and prevents MDA5-dependent immune activation. Cell Rep 36, 109500. 10.1016/j.celrep.2021.109500. 34380029

[29] Tang Q., Rigby R.E., Young G.R., Hvidt A.K., Davis T., Tan T.K., Bridgeman A., Townsend A.R., Kassiotis G., and Rehwinkel J. (2021). Adenosine-to-inosine editing of endogenous Z-form RNA by the deaminase ADAR1 prevents spontaneous MAVS-dependent type I interferon responses. Immunity 54, 1961–1975 e1965. 10.1016/j.immuni.2021.08.011. 34525337 PMC8459395

[30] Seet C.S., He C., Bethune M.T., Li S., Chick B., Gschweng E.H., Zhu Y., Kim K., Kohn D.B., Baltimore D., (2017). Generation of mature T cells from human hematopoietic stem and progenitor cells in artificial thymic organoids. Nat Methods 14, 521–530. 10.1038/nmeth.4237. 28369043 PMC5426913

[31] Xu L.D., and Ohman M. (2018). ADAR1 Editing and its Role in Cancer. Genes (Basel) 10. 10.3390/genes10010012. PMC6356570 30585209

[32] Cox C.V., Martin H.M., Kearns P.R., Virgo P., Evely R.S., and Blair A. (2007). Characterization of a progenitor cell population in childhood T-cell acute lymphoblastic leukemia. Blood 109, 674–682. 10.1182/blood-2006-06-030445. 17003368

[33] Song B., Shiromoto Y., Minakuchi M., and Nishikura K. (2022). The role of RNA editing enzyme ADAR1 in human disease. Wiley Interdiscip Rev RNA 13, e1665. 10.1002/wrna.1665. 34105255 PMC8651834

[34] Tan M.H., Li Q., Shanmugam R., Piskol R., Kohler J., Young A.N., Liu K.I., Zhang R., Ramaswami G., Ariyoshi K., (2017). Dynamic landscape and regulation of RNA editing in mammals. Nature 550, 249–254. 10.1038/nature24041. 29022589 PMC5723435

[35] Jain N., Lamb A.V., O’Brien S., Ravandi F., Konopleva M., Jabbour E., Zuo Z., Jorgensen J., Lin P., Pierce S., (2016). Early T-cell precursor acute lymphoblastic leukemia/lymphoma (ETP-ALL/LBL) in adolescents and adults: a high-risk subtype. Blood 127, 1863–1869. 10.1182/blood-2015-08-661702. 26747249 PMC4915808

[36] Girardi T., Vicente C., Cools J., and De Keersmaecker K. (2017). The genetics and molecular biology of T-ALL. Blood 129, 1113–1123. 10.1182/blood-2016-10-706465. 28115373 PMC5363819

[37] Liu Y., Easton J., Shao Y., Maciaszek J., Wang Z., Wilkinson M.R., McCastlain K., Edmonson M., Pounds S.B., Shi L., (2017). The genomic landscape of pediatric and young adult T-lineage acute lymphoblastic leukemia. Nat Genet 49, 1211–1218. 10.1038/ng.3909. 28671688 PMC5535770

[38] Ferrando A.A., Neuberg D.S., Staunton J., Loh M.L., Huard C., Raimondi S.C., Behm F.G., Pui C.H., Downing J.R., Gilliland D.G., (2002). Gene expression signatures define novel oncogenic pathways in T cell acute lymphoblastic leukemia. Cancer Cell 1, 75–87. 10.1016/s1535-6108(02)00018-1. 12086890

[39] Lu W.C., Xie H., Yuan C., Li J.J., Li Z.Y., and Wu A.H. (2020). Genomic landscape of the immune microenvironments of brain metastases in breast cancer. J Transl Med 18, 327. 10.1186/s12967-020-02503-9. 32867782 PMC7461335

[40] Passegue E., Jamieson C.H., Ailles L.E., and Weissman I.L. (2003). Normal and leukemic hematopoiesis: are leukemias a stem cell disorder or a reacquisition of stem cell characteristics? Proceedings of the National Academy of Sciences of the United States of America 100 Suppl 1, 11842–11849. 10.1073/pnas.2034201100. 14504387 PMC304096

[41] Bajaj J., Diaz E., and Reya T. (2020). Stem cells in cancer initiation and progression. J Cell Biol 219. 10.1083/jcb.201911053. PMC7039188 31874116

[42] Verovskaya E.V., Dellorusso P.V., and Passegue E. (2019). Losing Sense of Self and Surroundings: Hematopoietic Stem Cell Aging and Leukemic Transformation. Trends Mol Med 25, 494–515. 10.1016/j.molmed.2019.04.006. 31109796 PMC7657013

[43] Armstrong F., Brunet de la Grange P., Gerby B., Rouyez M.C., Calvo J., Fontenay M., Boissel N., Dombret H., Baruchel A., Landman-Parker J., (2009). NOTCH is a key regulator of human T-cell acute leukemia initiating cell activity. Blood 113, 1730–1740. 10.1182/blood-2008-02-138172. 18984862

[44] Park S.M., Cho H., Thornton A.M., Barlowe T.S., Chou T., Chhangawala S., Fairchild L., Taggart J., Chow A., Schurer A., (2019). IKZF2 Drives Leukemia Stem Cell Self-Renewal and Inhibits Myeloid Differentiation. Cell Stem Cell 24, 153–165 e157. 10.1016/j.stem.2018.10.016. 30472158 PMC6602096

[45] McCormack M.P., Shields B.J., Jackson J.T., Nasa C., Shi W., Slater N.J., Tremblay C.S., Rabbitts T.H., and Curtis D.J. (2013). Requirement for Lyl1 in a model of Lmo2-driven early T-cell precursor ALL. Blood 122, 2093–2103. 10.1182/blood-2012-09-458570. 23926305

[46] Komorowska K., Doyle A., Wahlestedt M., Subramaniam A., Debnath S., Chen J., Soneji S., Van Handel B., Mikkola H.K.A., Miharada K., (2017). Hepatic Leukemia Factor Maintains Quiescence of Hematopoietic Stem Cells and Protects the Stem Cell Pool during Regeneration. Cell Rep 21, 3514–3523. 10.1016/j.celrep.2017.11.084. 29262330

[47] Porath H.T., Carmi S., and Levanon E.Y. (2014). A genome-wide map of hyper-edited RNA reveals numerous new sites. Nat Commun 5, 4726. 10.1038/ncomms5726. 25158696 PMC4365171

[48] Carmi S., Borukhov I., and Levanon E.Y. (2011). Identification of widespread ultra-edited human RNAs. PLoS Genet 7, e1002317. 10.1371/journal.pgen.1002317. 22028664 PMC3197674

[49] Lamers M.M., van den Hoogen B.G., and Haagmans B.L. (2019). ADAR1: “Editor-in-Chief” of Cytoplasmic Innate Immunity. Front Immunol 10, 1763. 10.3389/fimmu.2019.01763. 31404141 PMC6669771

[50] Gannon H.S., Zou T., Kiessling M.K., Gao G.F., Cai D., Choi P.S., Ivan A.P., Buchumenski I., Berger A.C., Goldstein J.T., (2018). Identification of ADAR1 adenosine deaminase dependency in a subset of cancer cells. Nat Commun 9, 5450. 10.1038/s41467-018-07824-4. 30575730 PMC6303303

[51] Ahmad S., Mu X., and Hur S. (2021). The Role of RNA Editing in the Immune Response. Methods Mol Biol 2181, 287–307. 10.1007/978-1-0716-0787-9_17. 32729087

[52] Ishizuka J.J., Manguso R.T., Cheruiyot C.K., Bi K., Panda A., Iracheta-Vellve A., Miller B.C., Du P.P., Yates K.B., Dubrot J., (2019). Loss of ADAR1 in tumours overcomes resistance to immune checkpoint blockade. Nature 565, 43–48. 10.1038/s41586-018-0768-9. 30559380 PMC7241251

[53] Ota H., Sakurai M., Gupta R., Valente L., Wulff B.E., Ariyoshi K., Iizasa H., Davuluri R.V., and Nishikura K. (2013). ADAR1 forms a complex with Dicer to promote microRNA processing and RNA-induced gene silencing. Cell 153, 575–589. 10.1016/j.cell.2013.03.024. 23622242 PMC3651894

[54] Orlandi C., Barbon A., and Barlati S. (2012). Activity regulation of adenosine deaminases acting on RNA (ADARs). Mol Neurobiol 45, 61–75. 10.1007/s12035-011-8220-2. 22113393

[55] Yang S., Deng P., Zhu Z., Zhu J., Wang G., Zhang L., Chen A.F., Wang T., Sarkar S.N., Billiar T.R., and Wang Q. (2014). Adenosine deaminase acting on RNA 1 limits RIG-I RNA detection and suppresses IFN production responding to viral and endogenous RNAs. J Immunol 193, 3436–3445. 10.4049/jimmunol.1401136. 25172485 PMC4169998

[56] Pujantell M., Riveira-Munoz E., Badia R., Castellvi M., Garcia-Vidal E., Sirera G., Puig T., Ramirez C., Clotet B., Este J.A., and Ballana E. (2017). RNA editing by ADAR1 regulates innate and antiviral immune functions in primary macrophages. Sci Rep 7, 13339. 10.1038/s41598-017-13580-0. 29042669 PMC5645456

[57] Chen Y.G., and Hur S. (2022). Cellular origins of dsRNA, their recognition and consequences. Nat Rev Mol Cell Biol 23, 286–301. 10.1038/s41580-021-00430-1. 34815573 PMC8969093

[58] Zhang Z., and Carmichael G.G. (2001). The fate of dsRNA in the nucleus: a p54(nrb)-containing complex mediates the nuclear retention of promiscuously A-to-I edited RNAs. Cell 106, 465–475. 10.1016/s0092-8674(01)00466-4. 11525732

[59] De Bie J., Demeyer S., Alberti-Servera L., Geerdens E., Segers H., Broux M., De Keersmaecker K., Michaux L., Vandenberghe P., Voet T., (2018). Single-cell sequencing reveals the origin and the order of mutation acquisition in T-cell acute lymphoblastic leukemia. Leukemia 32, 1358–1369. 10.1038/s41375-018-0127-8. 29740158 PMC5990522

[60] Zhang J., Ding L., Holmfeldt L., Wu G., Heatley S.L., Payne-Turner D., Easton J., Chen X., Wang J., Rusch M., (2012). The genetic basis of early T-cell precursor acute lymphoblastic leukaemia. Nature 481, 157–163. 10.1038/nature10725. 22237106 PMC3267575

[61] Oshima K., Zhao J., Perez-Duran P., Brown J.A., Patino-Galindo J.A., Chu T., Quinn A., Gunning T., Belver L., Ambesi-Impiombato A., (2020). Mutational and functional genetics mapping of chemotherapy resistance mechanisms in relapsed acute lymphoblastic leukemia. Nat Cancer 1, 1113–1127. 10.1038/s43018-020-00124-1. 33796864 PMC8011577

[62] Vu L.P., Pickering B.F., Cheng Y., Zaccara S., Nguyen D., Minuesa G., Chou T., Chow A., Saletore Y., MacKay M., (2017). The N(6)-methyladenosine (m(6)A)-forming enzyme METTL3 controls myeloid differentiation of normal hematopoietic and leukemia cells. Nature medicine 23, 1369–1376. 10.1038/nm.4416. PMC5677536 28920958

[63] Graveley B.R., Brooks A.N., Carlson J.W., Duff M.O., Landolin J.M., Yang L., Artieri C.G., van Baren M.J., Boley N., Booth B.W., (2011). The developmental transcriptome of Drosophila melanogaster. Nature 471, 473–479. 10.1038/nature09715. 21179090 PMC3075879

[64] Lazzari E., Mondala P.K., Santos N.D., Miller A.C., Pineda G., Jiang Q., Leu H., Ali S.A., Ganesan A.P., Wu C.N., (2017). Alu-dependent RNA editing of GLI1 promotes malignant regeneration in multiple myeloma. Nat Commun 8, 1922. 10.1038/s41467-017-01890-w. 29203771 PMC5715072

[65] Bosticardo M., Pala F., Calzoni E., Delmonte O.M., Dobbs K., Gardner C.L., Sacchetti N., Kawai T., Garabedian E.K., Draper D., (2020). Artificial thymic organoids represent a reliable tool to study T-cell differentiation in patients with severe T-cell lymphopenia. Blood Adv 4, 2611–2616. 10.1182/bloodadvances.2020001730. 32556283 PMC7322962

[66] Guo Y., Dai Y., Yu H., Zhao S., Samuels D.C., and Shyr Y. (2017). Improvements and impacts of GRCh38 human reference on high throughput sequencing data analysis. Genomics 109, 83–90. 10.1016/j.ygeno.2017.01.005. 28131802

[67] Aken B.L., Ayling S., Barrell D., Clarke L., Curwen V., Fairley S., Fernandez Banet J., Billis K., Garcia Giron C., Hourlier T., (2016). The Ensembl gene annotation system. Database (Oxford) 2016. 10.1093/database/baw093. PMC4919035 27337980

[68] Dobin A., Davis C.A., Schlesinger F., Drenkow J., Zaleski C., Jha S., Batut P., Chaisson M., and Gingeras T.R. (2013). STAR: ultrafast universal RNA-seq aligner. Bioinformatics 29, 15–21. 10.1093/bioinformatics/bts635. 23104886 PMC3530905

[69] Robinson M.D., McCarthy D.J., and Smyth G.K. (2010). edgeR: a Bioconductor package for differential expression analysis of digital gene expression data. Bioinformatics 26, 139–140. 10.1093/bioinformatics/btp616. 19910308 PMC2796818

[70] Ritchie M.E., Phipson B., Wu D., Hu Y., Law C.W., Shi W., and Smyth G.K. (2015). limma powers differential expression analyses for RNA-sequencing and microarray studies. Nucleic Acids Res 43, e47. 10.1093/nar/gkv007. 25605792 PMC4402510

[71] Robinson M.D., and Oshlack A. (2010). A scaling normalization method for differential expression analysis of RNA-seq data. Genome Biol 11, R25. 10.1186/gb-2010-11-3-r25. 20196867 PMC2864565

[72] Wang L., Wang S., and Li W. (2012). RSeQC: quality control of RNA-seq experiments. Bioinformatics 28, 2184–2185. 10.1093/bioinformatics/bts356. 22743226

[73] Li B., and Dewey C.N. (2011). RSEM: accurate transcript quantification from RNA-Seq data with or without a reference genome. BMC Bioinformatics 12, 323. 10.1186/1471-2105-12-323. 21816040 PMC3163565

[74] Mortazavi A., Williams B.A., McCue K., Schaeffer L., and Wold B. (2008). Mapping and quantifying mammalian transcriptomes by RNA-Seq. Nat Methods 5, 621–628. 10.1038/nmeth.1226. 18516045 PMC13303166

[75] Ramaswami G., and Li J.B. (2014). RADAR: a rigorously annotated database of A-to-I RNA editing. Nucleic Acids Res 42, D109–113. 10.1093/nar/gkt996. 24163250 PMC3965033

[76] Kiran A., and Baranov P.V. (2010). DARNED: a DAtabase of RNa EDiting in humans. Bioinformatics 26, 1772–1776. 10.1093/bioinformatics/btq285. 20547637

[77] Zhao H., Sun Z., Wang J., Huang H., Kocher J.P., and Wang L. (2014). CrossMap: a versatile tool for coordinate conversion between genome assemblies. Bioinformatics 30, 1006–1007. 10.1093/bioinformatics/btt730. 24351709 PMC3967108

[78] Picardi E., and Pesole G. (2013). REDItools: high-throughput RNA editing detection made easy. Bioinformatics 29, 1813–1814. 10.1093/bioinformatics/btt287. 23742983

[79] Quinlan A.R., and Hall I.M. (2010). BEDTools: a flexible suite of utilities for comparing genomic features. Bioinformatics 26, 841–842. 10.1093/bioinformatics/btq033. 20110278 PMC2832824

